# Modifying the Siderophore Triacetylfusarinine C for Molecular Imaging of Fungal Infection

**DOI:** 10.1007/s11307-019-01325-6

**Published:** 2019-12

**Authors:** Piriya Kaeopookum, Dominik Summer, Joachim Pfister, Thomas Orasch, Beatrix E. Lechner, Milos Petrik, Zbynek Novy, Barbara Matuszczak, Christine Rangger, Hubertus Haas, Clemens Decristoforo

**Affiliations:** 1 Department of Nuclear Medicine, Medical University Innsbruck, Innsbruck, Austria; 2 Research and Development Division, Thailand Institute of Nuclear Technology, Nakhon Nayok, Thailand; 3 Division of Molecular Biology, Biocenter, Medical University Innsbruck, Innsbruck, Austria; 4 Institute of Molecular and Translational Medicine, Faculty of Medicine and Dentistry, Palacky University, Olomouc, Czech Republic; 5 Institute of Pharmacy, Pharmaceutical Chemistry, University of Innsbruck, Innsbruck, Austria

**Keywords:** Siderophores, Gallium-68, Triacetylfusarinine C, *Aspergillus fumigatus*, PET, Infection imaging

## Abstract

**Purpose:**

*Aspergillus fumigatus* produces the siderophore triacetylfusarinine C (TAFC) for iron acquisition which is essential for its virulence. Therefore, TAFC is a specific marker for invasive aspergillosis. We have shown previously that positron emission tomography (PET) imaging with [^68^Ga]TAFC exhibited excellent targeting properties in an *A. fumigatus* rat infection model. In this study, we aimed to prepare TAFC analogs modifying fusarinine C (FSC) by acylation with different carbon chain lengths as well as with charged substituents and investigated the influence of introduced substituents on preservation of TAFC characteristics *in vitro* and *in vivo*.

**Procedures:**

Fifteen TAFC derivatives were prepared and labeled with gallium-68. *In vitro* uptake assays were carried out in *A. fumigatus* under iron-replete as well as iron-depleted conditions and distribution coefficient was determined. Based on these assays, three compounds, [^68^Ga]tripropanoyl(FSC) ([^68^Ga]TPFC), [^68^Ga]diacetylbutanoyl(FSC) ([^68^Ga]DABuFC), and [^68^Ga]trisuccinyl(FSC) ([^68^Ga]FSC(suc)_3_), with high, medium, and low *in vitro* uptake in fungal cultures, were selected for further evaluation. Stability and protein binding were evaluated and *in vivo* imaging performed in the *A. fumigatus* rat infection model.

**Results:**

*In vitro* uptake studies using *A. fumigatus* revealed specific uptake of mono- and trisubstituted TAFC derivatives at RT. Lipophilicities as expressed by logD were 0.34 to − 3.80. The selected compounds displayed low protein binding and were stable in PBS and serum. Biodistribution and image contrast in PET/X-ray computed tomography of [^68^Ga]TPFC and [^68^Ga]DABuFC were comparable to [^68^Ga]TAFC, whereas no uptake in the infected region was observed with [^68^Ga]FSC(suc)_3_.

**Conclusions:**

Our studies show the possibility to modify TAFC without losing its properties and specific recognition by *A. fumigatus*. This opens also new ways for multimodality imaging or theranostics of fungal infection by introducing functionalities such as fluorescent dyes or antifungal moieties.

## Introduction

Due to the lack of sensitivity and specificity of current diagnostic methods, invasive fungal diseases are a major cause of high mortality rates (up to 50 % [1, 2]) and elevated healthcare costs in Europe [3]. *Aspergillus fumigatus* is the major cause for pulmonary fungal infections in immunocompromised patients including also transplant recipients and patients undergoing aggressive anti-cancer chemotherapy [4]. Early diagnosis of invasive pulmonary aspergillosis (IPA) is a key to improve survival rate. Various clinical tests and techniques as well as X-ray computed tomography (CT) have shown unsuccessful diagnosis in terms of specificity and sensitivity. Scintigraphic techniques such as single photon emission computer tomography (SPECT) and positron emission tomography (PET) have been applied for imaging fungal infections using non-specific radiotracers including [^99m^Tc]leucocytes, [^99m^Tc]peptides, [^99m^Tc]anti-granulocyte antibody, [^67^Ga]citrate (inflammation radiotracer), and even 2-deoxy-2-[^18^F]fluoro-D-glucose ([^18^F]FDG) [5]. These tracers have revealed only suboptimal characteristics for fungal detection.

**Fig. 1:**
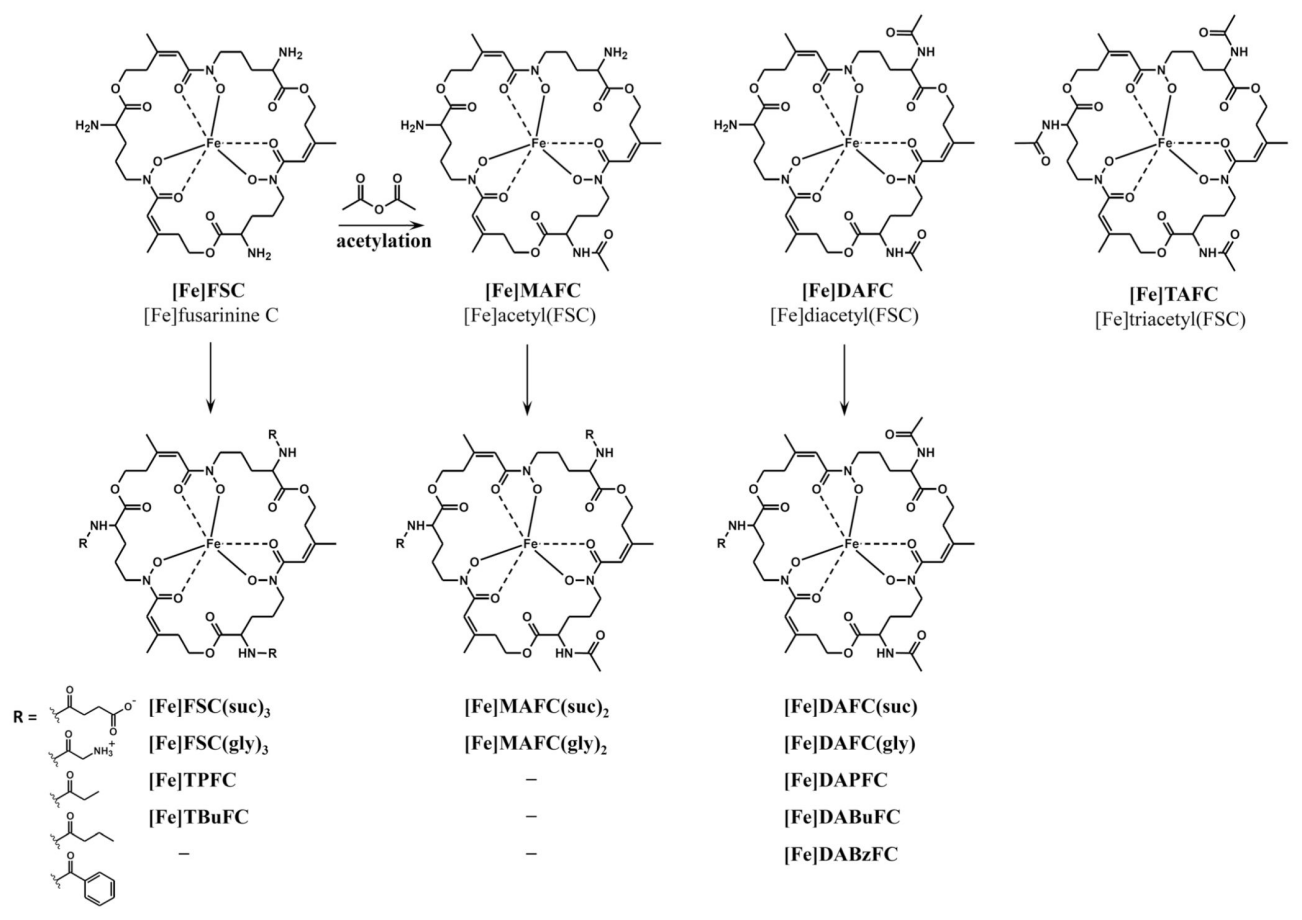
Syntheses of TAFC derivatives by conjugation of different substituents at free amino group(s) of FSC.

Iron is an essential nutrient for growth and virulence of pathogenic microorganisms [6]. Initiation of infection highly depends on the ability of microorganisms to use host-complexed iron. *A. fumigatus* lacks specific uptake systems for host iron sources and uses two high affinity iron uptake systems: reductive iron assimilation (Fe^2+^ specific) and siderophore-assisted iron acquisition (Fe^3+^ specific), but only the latter system is essential for virulence of *A. fumigatus* [7]. Siderophores, iron-sequestering compounds, are low molecular weight chelators with high affinity to iron (formation constants of 10^20^–10^50^) produced by fungi, bacteria, and some plants. *A. fumigatus* produces two hydroxamate-type siderophores, namely fusarinine C (FSC) and its *N*-acetylated derivative triacetylfusarinine C (TAFC), for extracellular iron acquisition [8, 9]. The chemical structures of both compounds are shown in Fig. 1. During infection, *A. fumigatus* faces an iron-starvation environment [10, 11] and excretes siderophores for “stealing” host iron. After chelation of iron, siderophore-iron complexes are taken up through siderophore-iron transporters (SITs), which are members of a subfamily of major facilitator protein superfamily [12]. In *A. fumigatus*, MirB has been identified as a specific transporter for TAFC whereas the FSC transporter still remains to be identified [13, 14]. The MirB transporter is highly upregulated during infection and is found in *A. fumigatus*, but not in bacteria and several other fungal strains. Furthermore, human lacks SITs such as MirB and therefore specific substrates do not interact with human cellular systems. These characteristics promote targeted diagnosis of *A. fumigatus* infection *via* the MirB transporter using TAFC as the vector molecule.

PET has been widely used for molecular imaging due to the high-intensity images, limitless depth of penetration, and providing quantitative data. Among positron emitter isotopes, gallium-68 (Ga-68) is the most attractive nuclide for radiolabeling of siderophores [15]. Ga^3+^ has an equal charge and a comparable radius to ferric ion (Fe^3+^) allowing displacement of iron by Ga-68. Moreover, gallium-68 has a half-life of 68 min exhibiting very low radiation burden to patients. Furthermore, it can be obtained from ^68^Ge/^68^Ga generator systems, therefore easy, accessible, simple in use, and relatively inexpensive.

In our previous works, we have demonstrated that different siderophores can be labeled with ^68^Ga [16]. We have also shown that [^68^Ga]TAFC and [^68^Ga]ferrioxamine E ([^68^Ga]FOXE) are able to detect *A. fumigatus* infection in a rat pulmonary aspergillosis model using PET imaging [17, 18] and [^68^Ga]TAFC is more specific to *A. fumigatus in vitro* [19]. [^68^Ga]TAFC exhibits very rapid renal elimination, resulting in a short-term bioavailability. Chemically modifying TAFC would potentially allow adjusting pharmacokinetic properties and introducing functionalities such as fluorescent dyes or even therapeutic moieties. In this paper, we describe chemical modifications of TAFC (see Fig. 1), [^68^Ga]-radiolabeling, and *in vitro* characterization of respective gallium complexes. We have also investigated the influence of TAFC modifications on the recognition by the specific siderophore system of *A. fumigatus* and report the biodistribution as well as PET/CT images of selected compounds in comparison to [^68^Ga]TAFC.

## Materials and Methods

### Chemicals

All commercially available chemicals were obtained as analytical grade and used without further purification. The ^68^Ge/^68^Ga generator (IGG100) was purchased from Eckert & Ziegler Strahlenund Medizintechnik AG (Berlin, Germany) with a nominal activity of 1850 MBq and was eluted with 0.1 M HCl solution (Rotem Industries Ltd., Beer-Sheva, Israel) using the fractionated elution approach.

### Fungal Strains and Growth Conditions

For *in vitro* uptake studies, *A. fumigatus wildtype* (ATCC46645) was used. Fungal strain was cultured at 37 °C in *Aspergillus* minimal medium (AMM) containing 1 % (*m*/*v*) glucose as carbon source, 20 mM glutamine as nitrogen source, salts, and trace elements [20]. Iron-sufficient media were prepared by adding FeSO_4_ to a final concentration of 30 μM. For iron-deficient conditions, supplementation of iron was omitted. Iron starvation was verified by the detection of extracellular siderophore production which is suppressed by iron supplementation.

For siderophore utilization growth assays, *A. fumigatus* mutant strain Δ*sidA/*Δ*ftrA* [7], which can grow only in the presence of externally added iron-siderophores or at high iron concentrations, was used. The fungal strain was inoculated in iron-deficient culture as described above.

### Preparation of [Fe]Fusarinine C from *A. fumigatus*

[Fe]fusarinine C ([Fe]FSC) was extracted from the *A. fumigatus* mutant strain *ΔsidG*, which lacks the conversion of FSC to TAFC [9]. Fungi were cultured under iron-depleted condition as described by Schrettl, et al. [9] with slightly changed incubation time and extraction method as previously published [21].

### Synthesis

The syntheses of TAFC derivatives are illustrated in Fig. 1 and described in ELECTRONIC Supplementary Material (ESM). Briefly, amino group(s) of [Fe]FSC were acetylated to give acetyl[Fe]FSC ([Fe]MAFC), diacetyl[Fe]FSC ([Fe]DAFC), and triacetyl[Fe]FSC ([Fe]TAFC) which were then used as the starting materials of further syntheses. Propanoyl-, butanoyl-, and benzoylchloride were used for acylations to obtain tripropanoyl[Fe]FSC ([Fe]TPFC), propanoyl[Fe]DAFC ([Fe]DAPFC), tributanoyl[Fe]FSC ([Fe]TBuFC), butanoyl[Fe]DAFC ([Fe]DABuFC), and benzoyl[Fe]DAFC ([Fe]DABzFC). Negative and positive charged moieties were introduced to [Fe]DAFC, [Fe]MAFC, and [Fe]FSC by reaction with succinic anhydride and Fmocgly-OH, respectively, resulting in succinyl[Fe]DAFC ([Fe]DAFC(suc)), disuccinyl[Fe]MAFC ([Fe]MAFC(suc)_2_), trisuccinyl[Fe]FSC ([Fe]FSC(suc)_3_), glycyl[Fe]DAFC ([Fe]DAFC(gly)), diglycyl[Fe]MAFC ([Fe]MAFC(gly)_2_), and triglycyl[Fe]FSC ([Fe]FSC(gly)_3_). Iron-free siderophores were finally accomplished by demetalation using disodium-ethylenediaminetetraacetic acid (see ESM).

### Radiolabeling

To 10 μg of siderophore, 200 μl [^68^Ga]GaCl_3_ (approx. 35–40 MBq) in 0.1 M HCl (fractionated elution) was added. The pH of solution was adjusted to 4.5 by adding 40 μl of 1.1 M sodium acetate (NaOAc). The labeling mixture was incubated at 75 °C for 10 min. Radiochemical yield (%RCY) of labeled [^68^Ga]siderophore was determined using a reversed-phase high-performance liquid chromatography (RP-HPLC) and/or radio-instant thin layer chromatography (radio-ITLC).

### *In Vitro* Characterization

#### Distribution Coefficient (logD)

[^68^Ga]siderophore (4 μM) in 500 μl phosphate-buffered saline (PBS), pH 7.4, was added to 500 μl octanol. The mixture was vortexed at 1400 rpm for 15 min and subsequently centrifuged at 2000 rcf for 2 min. Aliquots of aqueous and octanol phases were collected and measured in a gamma counter (a 2480 Automatic Gamma Counter Wizard^2^ 3″; PerkinElmer, Waltham, MA, USA). The logD values were calculated from the obtained data (*n* = 5).

#### Stability

Stability of selected siderophores, [^68^Ga]TAFC, [^68^Ga]TPFC, [^68^Ga]DABuFC, and [^68^Ga]FSC(suc)_3_, was evaluated in PBS pH 7.4 and fresh human serum after 0, 30, 60, and 120 min incubation. [^68^Ga]siderophore (50 μl, 2 nmol) was incubated in 450 μl PBS or human serum at 37 °C. At each time point, aliquot of PBS was injected directly to RP-HPLC whereas serum aliquot was mixed with MeOH, vortexed, and centrifuged at 20,000 rcf for 2 min. Supernatant was diluted with H_2_O and subsequently analyzed by RP-HPLC. The stability was presented as %RCP of radiotracer (*n* = 3).

#### Protein Binding

Protein binding abilities of [^68^Ga]TAFC, [^68^Ga]TPFC, [^68^Ga]DABuFC, and [^68^Ga]FSC(suc)_3_ were studied by incubating radiotracers in human serum at 37 °C for 30, 60, and 120 min. At selected time points, 25 μl of serum aliquots was applied on size exclusion spin columns MicroSpin™ G-50 (GE Healthcare, Vienna, Austria). Columns were centrifuged at 2000 rcf for 2 min to separate protein bound radiotracer (eluate) from non-protein bound radiotracer (column). Protein binding ability was determined by measuring activities of column and eluate in the gamma counter. The results were displayed as % protein bound to total activity applied (*n* = 3).

### *In Vitro* Uptake Assay in *A. fumigatus*

Uptake assays were performed in either iron-deficient or iron-sufficient *A. fumigatus wildtype* cultures. In a 96-well MultiScreen Filter Plates HTS (1 μm glass fiber filter, Merck Millipore, Darmstadt, Germany), 180 μl of *A. fumigatus* suspended in iron-replete or iron-depleted medium was added. [^68^Ga]siderophores (approx. 1 × 10^5^ cpm, 80 nM) were incubated with either PBS pH 7.4 (total series) or blocking solution (10 μM [Fe]TAFC) (non-specific series) at RT for 45 min in quadriplicates. The incubation was interrupted by suction of the medium and the plate was washed twice with ice-cold TRIS-buffered saline (10 mM, pH 7.3). The filters were isolated and the remaining activities were measured in the gamma counter. The uptake was calculated as % uptake of total activity added; each assay was repeated three times. To normalize uptake variations between different fungal cultures, the uptake of each [^68^Ga]siderophore was expressed as the ratio to [^68^Ga]TAFC uptake from the same experiment. The mean values of three different assays were used to quantitatively compare the difference in uptake between the compounds.

### *In Vitro* Uptake Assay in *A. terreus*

To confirm the specificity of uptake of TAFC derivatives by *A. fumigatus via* the MirB transporter, *A. terreus*, which lacks the MirB transporter, was used for *in vitro* uptake assay. *A. terreus* was grown in either iron-deficient or iron-sufficient cultures. Uptake studies were performed as described for *A. fumigatus* and expressed as % uptake of total activity added.

### Siderophore Utilization Growth Assay of *A. fumigatus*

To investigate the ability or inability of *A. fumigatus* to take up and utilize [Fe]siderophores as iron source, a growth assay with the *A. fumigatus* mutant strain Δ*sidA/*Δ*ftrA* was employed. In a 24-well plate, aliquots of 10^3^ conidia of mutant strain were point inoculated on 1 ml of AMM containing 0.1, 1, 10, 50, or 100 μM of [Fe]siderophore and incubated at 37 °C for 48 h. For the control, fungi were grown in iron-limited AMM without adding [Fe]siderophore.

### Animal Experiments

#### Biodistribution Studies

Animal experiments were conducted in compliance with the regulation and guidelines of the Austrian animal protection laws and with the approval of Austrian Ministry of Science (BMWFW-66.011/0161-WF/V/3b/2016).

#### Small Animal PET/CT Imaging

Animal experiments were conducted in accordance with the regulation and guidelines of the Czech Animal Protection Act (No. 246/1992) and with the approval of the Czech Ministry of Education Youth and Sports (MSMT-21275/2016-2), and the institutional Animal Welfare Committee of the Faculty of Medicine and Dentistry of Palacky University in Olomouc.

#### Biodistribution in BALB/c Mice

Biodistribution of [^68^Ga]TAFC, [^68^Ga]TPFC, [^68^Ga]DABuFC, and [^68^Ga]FSC(suc)_3_ was evaluated in healthy BALB/c mice. A group of 3 mice (female, 6 weeks) were injected intravenously with [^68^Ga]TAFC, [^68^Ga]TPFC, [^68^Ga]DABuFC, or [^68^Ga]FSC(suc)_3_ (2 MBq/mouse, 1–2 μg siderophore) into the lateral tail vein. At 90 min post injetion (p.i.), mice were sacrified by cervical dislocation. Organs and tissues (blood, spleen, pancrease, stomach, intestine, kidneys, liver, heart, lung, muscle, femur) were collected and weighed. Radioactivities accumulated by tissues were measured in the gamma counter. The obtained values were expressed as the percentage of injected dose per gram tissue (%ID/g). Urine sample from each animal was injected to RP-HPLC for analysis of urinary metabolic profile of [^68^Ga]siderophore.

#### Small Animal PET/CT Imaging in Rat IPA Model

PET and CT images were acquired with an Albira PET/SPECT/CT small animal imaging system (Bruker Biospin Corporation, Woodbridge, CT, USA). *A. fumigatus* infection in rats was established as described by Petrik, et al. [18]. A group of 3 female Lewis rats (2–3 months old), healthy or lung infected, were injected retro-orbitally (r.o.) with [^68^Ga] TAFC, [^68^Ga]TPFC, [^68^Ga]DABuFC, or [^68^Ga]FSC(suc)_3_ in a dose of ~ 6 MBq corresponding to 2 μg of siderophore per rat. Animals were anesthetized with isoflurane (FORANE, Abbott Laboratories, Abbott Park, IL, USA) (2 % flow rate) and positioned prone head first in the Albira system. PET/CT imaging was started 45 min p.i. with a 10-min PET (FOV 148 mm axial) and a subsequent triple CT scanning (FOV 3 × 65 mm axial, 45 kVp, 400 μA, at 400 projections). For reconstruction, Albira software (Bruker Biosoin Corporation, Woodbridge, CZ, USA) was used involving filtered backprojection and MLEM algorithms. PMOD software (PMOD Technologies Ltd., Zurich, Switzerland) was used to analyze and view the reconstructed images.

### Statistical Analysis

The *in vitro* uptake data of each [^68^Ga]siderophore and [^68^Ga]TAFC from the same experiment were compared using unpaired *t* test (level of significance, *P* < 0.01). Analysis was performed using Microsoft Office Excel 2010 program.

## Results

### Synthesis of Siderophores

Iron-containing siderophores were produced in yields between 38 and 67 %. Acetylation of [Fe]FSC yielded a mixture of [Fe]MAFC, [Fe]DAFC, and [Fe]TAFC with a total yield of 63 %. The acyl and succinyl siderophores from direct conjugation were received in yields between 52 and 65 %, whereas glycyl siderophores requiring an additional deprotection step resulted in somewhat lower yields 38–51 %. Removal of iron and subsequent RP-HPLC purification resulted in yields between 43 and 67 %. Characterization by MALDI-TOF MS confirmed the theoretically calculated masses (see ESM). Yields of both iron-containing and iron-free siderophores are summarized in Table 1.

### Ga-68 Labeling of Siderophores

At the stated conditions (10 μg siderophore; pH 4.5; 75 °C; 10 min incubation), [^68^Ga]siderophores were obtained in almost quantitative %RCY (> 99 %) for most compounds and used without further purification. For [^68^Ga]DABzFC, [^68^Ga]DAFC (gly), [^68^Ga]MAFC(gly)_2_, and [^68^Ga]FSC(gly)_3_, lower RCY between 7 and 94 % were achieved. For subsequent characterization, these labeled compounds were purified *via* solid phase extraction resulting in RCP > 95 %. Radiochemical yields (%RCY) of [^68^Ga]-labeled compounds are presented in Table 1.

### Distribution Coefficient, Protein Binding, and Stability Studies

Lipophilicity as expressed by the logD value varied between 0.34 and − 3.80 indicating a wide variation of lipophilicity (see Table 1). All compounds were stable after radiolabeling for > 2 h. Protein binding and stability over time for four selected compounds ([^68^Ga]-TAFC, -TPFC, -DABuFC, -FSC(suc)_3_) are shown in Table 2.

### *In Vitro* Uptake of [^68^Ga]Siderophores

**Fig. 2:**
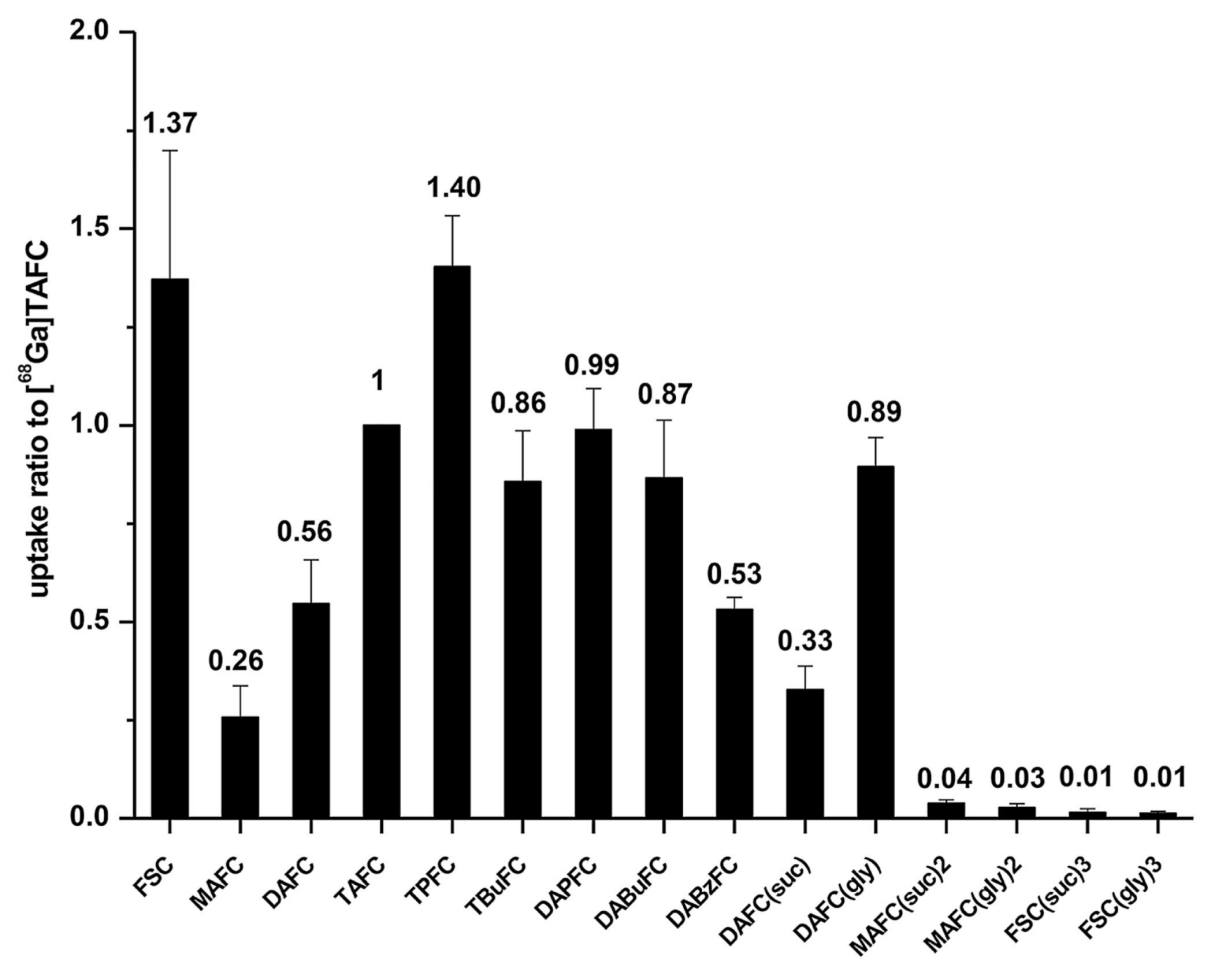
*In vitro* uptake of [^68^Ga]siderophores in *A. fumigatus* under iron-starvation condition at 37 °C for 45 min. Normalized uptakes are expressed as ratio to [^68^Ga]TAFC uptake from the same experiment (*n* = 3). Uptake under iron-starvation condition with TAFC blocking and under iron-replete condition, which were used as controls, was < 0.5 % of total activity added (data not shown).

The certain fluctuation of % uptake of [^68^Ga]siderophore in *A. fumigatus cultures* from different experiments was observed indicating variation in expression of SITs dependent on individual iron-starvation conditions. Nonetheless, reproducible uptake ratios could be obtained by normalizing to [^68^Ga]TAFC as demonstrated in Fig. 2. The relative uptake varied from 0.01 to 1.40 for different TAFC derivatives. [^68^Ga]-TBuFC, -DAPFC, -DABuFC, and -DAFC(gly) showed comparable uptake to [^68^Ga]TAFC with values around 1; medium uptake was observed for [^68^Ga]-MAFC, -DAFC, -DABzFC, and -DAFC(suc) between 0.26 and 0.55. Very low uptake ratio was found for [^68^Ga]-MAFC(suc)_2_, -MAFC(gly)_2_, -FSC(suc)_3_, and -FSC(gly)_3_ with less than 0.05. Higher uptake was observed in [^68^Ga]-TPFC and -FSC with approximate values of 1.4; however, only [^68^Ga]TPFC was considered for further experiments since -FSC is most likely not transported by the MirB but other SITs [12]. This was confirmed by uptake studies using *A. terreus*, which lacks the MirB transporter. *A. terreus* showed high uptake of [^68^Ga]FSC (> 30 %) whereas all other compounds exhibited very low values below 2 % of total activity added (see Suppl. Fig. 1 in ESM).

### Utilization of Siderophore by *A. fumigatus*

**Fig. 3:**
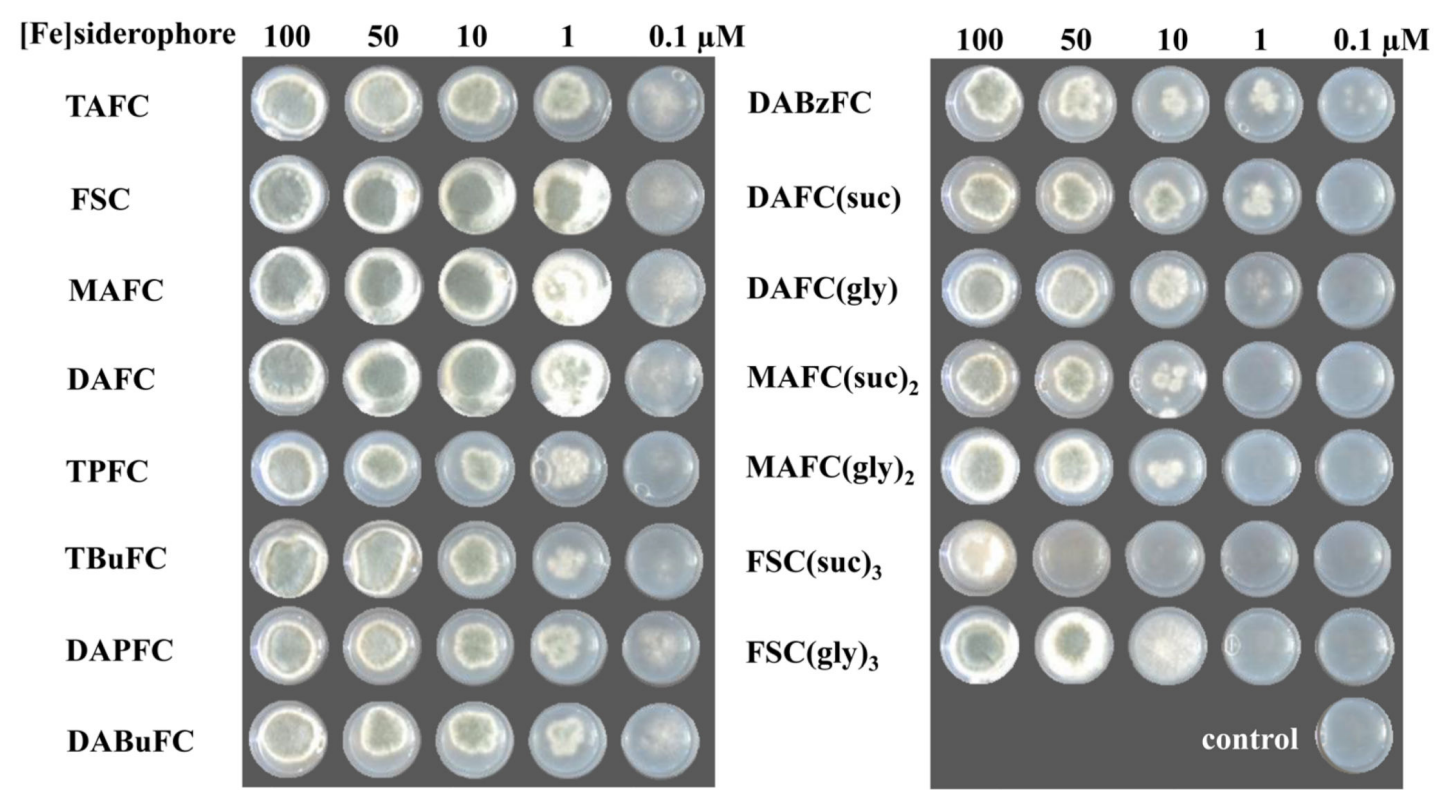
Utilization of [Fe]siderophores by the *A. fumigatus* mutant strain *ΔsidA/ΔftrA* lacking siderophore biosynthesis and reductive iron acquisition. The picture was taken after incubation at 37 °C for 48 h at different concentrations (μM) of [Fe]siderophore in AMM.

A double gene deletion mutant strain *A. fumigatus*, Δ*sidA/*Δ*ftrA*, which lacks siderophore biosynthesis and reductive iron assimilation, is unable to grow in the absence of siderophore or Fe^2+^ concentration above 2 mM. Therefore, this strain was used to investigate the utilization of external supplied [Fe]siderophores for its growth. This assay showed the utilization of all studied siderophores by *A. fumigatus* dependent on the concentration of the different [Fe]siderophores. Nevertheless, no significant difference in growth was observed at high concentration (50–100 μM), except for FSC(suc)_3_ that showed a sporulation defect at 100 μM but no growth at 50 μM (Fig. 3). After 48 h incubation, the growth of *A. fumigatus* was supported by [Fe]-FSC, -MAFC, and -DAFC already at very low concentrations (0.1 μM). Medium degree of utilization was found in [Fe]-TAFC, -TPFC, -TBuFC, -DAPFC, -DABuFC, and -DABzFC. Compared to [Fe]TAFC, these siderophores promoted the growth in agreement with behaviors observed in the *in vitro* uptake assay. For [Fe]-DAFC(suc), -DAFC(gly), -MAFC(suc)_2_, -MAFC(gly)_2_, and -FSC(gly)_3_, *A. fumigatus* growth was supported in low degree and required higher concentration of [Fe]siderophores (1 μM). The lowest level of growth was observed in [Fe]FSC(suc)_3_, indicating that it can be used as iron source for the *A. fumigatus* only in an extremely high concentration, correlating well with its low short-term uptake when radiolabeled with Ga-68.

### Biodistribution in BALB/c Mice

**Fig. 4:**
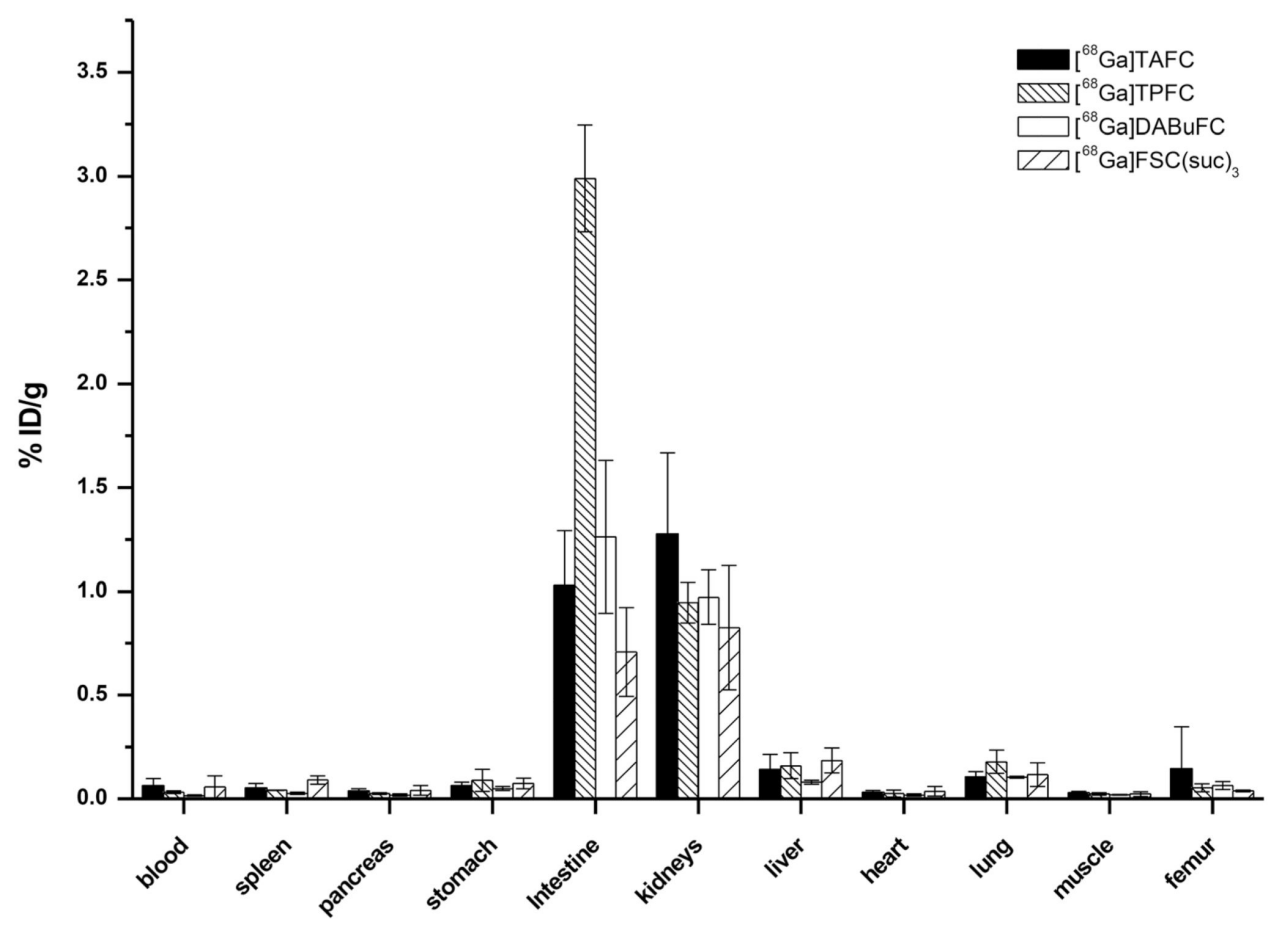
Biodistribution in normal BALB/c mice at 90 min p.i. of [^68^Ga]TAFC, [^68^Ga]TPFC, [^68^Ga]DABuFC, and [^68^Ga]FSC(suc)_3_ (*n* = 3). Tissue uptake values are presented as percentages of injected dose per gram of tissue (%ID/g).

[^68^Ga]TAFC was applied as a standard while [^68^Ga]TPFC, [^68^Ga]DABuFC, and [^68^Ga]FSC(suc)_3_ were chosen as candidates of PET imaging agents due to its higher, comparable, and lower *in vitro* uptake compared to [^68^Ga]TAFC, respectively. In healthy BALB/c mice at 90 min p.i., [^68^Ga]TAFC, [^68^Ga]TPFC, [^68^Ga]DABuFC, and [^68^Ga]FSC(suc)_3_ revealed rapid renal excretion, low blood levels (< 0.1 %ID/g), and minimal retention in other organs. In comparison with [^68^Ga]TAFC, [^68^Ga]-TPFC, -DABuFC, and -FSC(suc)_3_ displayed similar radioactivity accumulation in almost all tissues as illustrated in Fig. 4. Slightly lower retention was observed only in the blood, spleen, and pancreas for [^68^Ga]DABuFC and in the intestine and kidneys for [^68^Ga]FSC(suc)_3_. Higher uptake was found in intestine for [^68^Ga]TPFC but still lower than 3 %ID/g. In urine, only intact [^68^Ga]TAFC, [^68^Ga]TPFC, [^68^Ga]DABuFC, and [^68^Ga]FSC(suc)_3_ were detected.

### Small Animal PET/CT Imaging in Rat IPA Model

**Fig. 5:**
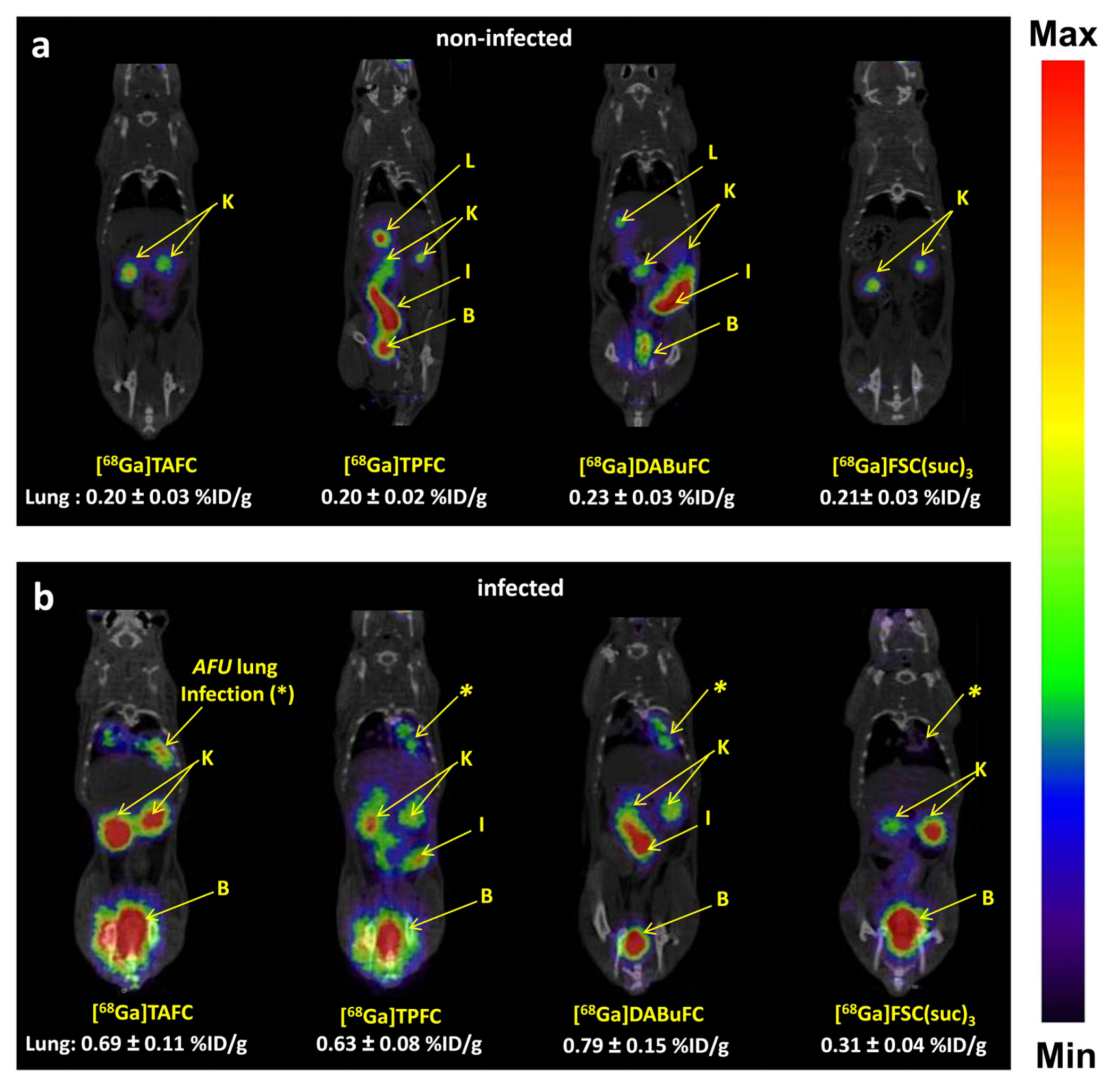
Static microPET/CT images of [^68^Ga]TAFC, [^68^Ga]TPFC, [^68^Ga]DABuFC, and [^68^Ga]FSC(suc)_3_ at 45 min p.i.: fused PET/CT coronal slices (prone position) in **a** non-infected Lewis rats and **b** IPA Lewis rats (approx. 6 MBq injected dose; B, bladder; I, intestine; K, kidney; L, liver).

PET/CT images in healthy Lewis rats confirmed the *in vivo* properties found in biodistribution studies of healthy BALB/c mice. All studied compounds, [^68^Ga]TAFC, [^68^Ga]TPFC, [^68^Ga]DABuFC, and [^68^Ga]FSC(suc)_3_, were excreted rapidly *via* the urinary system with very low background activity in all tissues except the kidneys. [^68^Ga]TPFC and [^68^Ga]DABuFC showed certain hepatobiliary excretion with some residual activity in the liver and intestine. No uptake in the lung region was observed in non-infected animals (Fig. 5a).

For *in vivo* imaging in a rat IPA model, visible infected lungs with moderate intensity were traced by [^68^Ga]TAFC, [^68^Ga]TPFC, and [^68^Ga]DABuFC (0.6–0.8 %ID/g) revealing the recognition, selectivity, and specificity of these [^68^Ga]siderophores to *A. fumigatus.* On the other hand, [^68^Ga]FSC(suc)_3_ showed uptake in infected lung regions (0.3 %ID/g) comparable to non-infected tissue (see Fig. 5b).

## Discussion

[^68^Ga]TAFC has shown excellent targeting properties for *A. fumigatus* infections *in vitro* and *in vivo* [17, 18], allowing molecular imaging of fungal infection using PET. TAFC is the main siderophore produced and excreted by *A. fumigatus* for iron utilization and growth. It is unknown which structural elements are required to achieve selective recognition of [Fe] complexes by MirB. Modification of TAFC may allow to particularly adjusting pharmacokinetics to optimize targeting of fungal infection *in vivo*, but also to introduce functionalities into the molecule for a variety of novel applications including theranostics of fungal infections, which was recently proposed for bacterial infections using multivalent siderophore-DOTAM conjugates [22]. We have previously shown that starting from FSC, the deacetylated variant of TAFC, selective substitution, and chemical modifications are possible. Mono-, di-, and trisubstituted FSC derivatives were prepared by acylation of the free amines [21, 23, 24]. We utilized this approach to introduce targeting vectors and even fluorescent dyes as basis for the preparation of novel Ga-68 and Zr-89 radiopharmaceuticals for oncological applications. In this study, we aimed to reveal some structure-activity relationships of fungal recognition by selective acylation of one, two, or three amine groups of the FSC molecule. We chose to introduce longer carbon substituents (propanoyl, butanoyl, benzoyl) instead of acetyl as well as positively and negatively charged moieties by using glycyl and succinyl substituents, respectively. Besides mono-substitution of the acetyl, we also prepared selected di- and trisubstituted derivatives. All compounds could be prepared straight forward in reasonable yields by starting from the deacetylated compounds, i.e., DAFC for mono-, MAFC for di-, and FSC for trisubstituents, protected by using the respective [Fe] complexes. After removal of iron, all compounds could be radiolabeled with gallium-68 in quantitative yields. Only for a couple of derivatives, radiolabeling was less efficient for unknown reasons, which required purification before further use. The tested compounds showed a wide variety of lipophilicity and various charges depending on the substituents.

In initial uptake assays, most Ga-68 compounds showed a specific uptake by *A. fumigatus*, when grown under iron starvation, indicating recognition by siderophore transporters. There was no indication for any changes in transporter recognition, except for FSC, which is known to be utilized not only by MirB. However, the efficiency in uptake was highly variable. Highest uptake was found for trisubstituted of propanoyl (tripropanoyl(FSC)) while tributanoyl(FSC) revealed a lower uptake which could be related to its increased lipophilicity. Comparable uptakes were observed in mono-substituted, i.e., DAFC derivatives, with a slight decrease in uptake with higher lipophilicity (e.g., DABzFC) or when a negative charge was introduced (DAFC(suc)). TAFC derivatives with di- and trisubstitutions with charged moieties resulted in loss of specific uptake by *A. fumigatus*. These uptake trends were confirmed by growth assays using an *A. fumigatus* mutant lacking siderophore production and reductive iron assimilation. This mutant only grows in the presence of external siderophores. Also in this assay, mono-substituted TAFC compounds showed comparable or only slightly impaired growth promotion as compared to TAFC, whereas with additional substitution, growth promotion was significantly impaired. This indicates that by modifying TAFC, not only uptake can be preserved but also *A. fumigatus* can utilize these modified siderophores as source of iron.

*In vivo* behavior was tested for three selected compounds ([^68^Ga]TPFC, [^68^Ga]DABuFC, [^68^Ga]FSC(suc)_3_) in comparison to [^68^Ga]TAFC. In both biodistribution and imaging experiments in healthy animals, [^68^Ga]TPFC, [^68^Ga]DABuFC, and [^68^Ga]FSC(suc)_3_ revealed comparative *in vivo* behaviors. As expected, the somewhat more lipophilic compounds [^68^Ga]TPFC and [^68^Ga]DABuFC showed higher trend towards hepatobiliary and intestinal excretion. This was only seen in PET/CT studies performed at 45 min p.i., whereas the difference in biodistribution studies at later time points (90 min p.i.) was less pronounced. However, rapid blood clearance and washout from normal tissue with renal excretion of the intact [^68^Ga]siderophores, as analyzed by HPLC, was observed. Imaging in lung infection model exhibited comparable contrast for [^68^Ga]TPFC and [^68^Ga]DABuFC and no visible infected lung for [^68^Ga]FSC(suc)_3_, which matches the *in vitro* uptake results. Nonetheless, interference from accumulated activity in intestine *via* intestinal excretion was only seen in [^68^Ga]TPFC and [^68^Ga]DABuFC due to their higher lipophilicities. Therefore, these siderophores are not more efficient than TAFC itself in terms of imaging contrast but evidence the possibility of modifying TAFC without losing specific recognition by *A. fumigatus*.

## Conclusions

Overall, we could show in this paper that modification of TAFC is possible without losing major properties of this promising siderophore for molecular imaging of *A. fumigatus* infections. Selective replacement of one acetyl group results in compounds that can be easily radiolabeled with gallium-68 and show high uptake by *A. fumigatus* and specific recognition by the MirB siderophore transporter as well as high stability both *in vitro* and *in vivo*. This knowledge enables us to design compounds with varying pharmacokinetics potentially improving targeting properties, but also to introduce functionalities such as fluorescent dyes to combine optical imaging with PET or to introduce moieties with antifungal activity opening new ways for theranostics also in the field of infectious diseases.

## Supplementary Material

Electronic supplementary material The online version of this article (https://doi.org/10.1007/s11307-019-01325-6) contains supplementary material,which is available to authorized users.

ESM 1

Supplementary Figure 1

## Figures and Tables

**Table 1:** Radiochemical yield (%RCY), distribution coefficient (logD) of [^68^Ga]siderophores, and synthetic yield of siderophores

Siderophore	% yield		[^68^Ga]siderophore	
	
	+[Fe]	−[Fe]	%RCY *(n = 3)*	logD
FSC	*NA*	55	99.9	− 2.83 ± 0.16
MAFC	8	58	99.8	− 2.67 ± 0.08
DAFC	32	57	99.8	− 2.34 ± 0.09
TAFC	25	67	99.9	− 2.08 ± 0.02
TPFC	52	63	99.8	− 0.83 ± 0.02
TBuFC	53	54	99.8	0.34 ± 0.01
DAPFC	65	61	99.8	− 1.61 ± 0.07
DABuFC	53	50	99.8	− 1.21 ± 0.11
DABzFC	57	53	75.3	− 0.38 ± 0.02
DAFC(suc)	55	59	99.7	− 3.29 ± 0.14
DAFC(gly)	51	61	93.7	− 2.52 ± 0.02
MAFC(suc)_2_	58	60	99.7	− 3.59 ± 0.11
MAFC(gly)_2_	44	56	13.5	− 2.91 ± 0.07
FSC(suc)_3_	64	58	99.8	− 3.80 ± 0.06
FSC(gly)_3_	38	43	7.9	− 2.82 ± 0.06

**Table 2:** Protein binding, stabilities in PBS (pH 7.4), and human serum of [^68^Ga]TAFC, [^68^Ga]TPFC, [^68^Ga]DABuFC, and [^68^Ga]FSC(suc)_3_

[^68^Ga]siderophore	Incubation time (min)	Protein binding (%) (*n* = 3)	Stability in PBS (%) (*n* = 3)	Stability in serum (%) (*n* = 3)
[^68^Ga]TAFC	30	2.54 ± 1.01	99.89 ± 0.02	99.48 ± 0.29
	60	3.55 ± 0.68	99.83 ± 0.13	99.46 ± 0.09
	120	2.96 ± 0.33	99.73 ± 0.03	99.41 ± 0.19
[^68^Ga]TPFC	30	2.31 ± 0.57	99.90 ± 0.02	99.67 ± 0.19
	60	3.39 ± 0.32	99.86 ± 0.04	99.56 ± 0.23
	120	2.65 ± 0.66	99.79 ± 0.11	99.54 ± 0.25
[^68^Ga]DABuFC	30	2.63 ± 1.03	99.87 ± 0.07	99.58 ± 0.04
	60	2.92 ± 1.11	99.85 ± 0.08	99.59 ± 0.20
	120	3.61 ± 1.19	99.79 ± 0.14	99.60 ± 0.14
[^68^Ga]FSC(suc)_3_	30	2.53 ± 0.63	99.85 ± 0.07	99.59 ± 0.08
	60	2.96 ± 0.21	99.77 ± 0.12	99.43 ± 0.17
	120	2.60 ± 0.62	99.72 ± 0.09	99.36 ± 0.08
