# Supplementary material for: Modifying the Siderophore Triacetylfusarinine C for Molecular Imaging of Fungal Infection
Source: Mol Imaging Biol. Author manuscript; Available in PMC 2020 Mar 1. (PMC6877352; doi:10.1007/s11307-019-01325-6)
Supplement: ESM 1 [file EMS82694-supplement-ESM_1.docx]

**Supplemental Material**

**Modifying the Siderophore Triacetylfusarinine C for Molecular Imaging of Fungal Infection**

Piriya Kaeopookum,^1,5^ Dominik Summer,^1^ Joachim Pfister,^1^ Thomas Orasch,^2^ Beatrix E. Lechner,^2^ Milos Petrik,^3^ Zbynek Novy,^3^ Barbara Matuszczak,^4^ Christine Rangger,^1^ Hubertus Haas,^2^ Clemens Decristoforo^1^

^1^*Department of Nuclear Medicine, Medical University Innsbruck, Innsbruck, Austria*

^2^*Division of Molecular Biology, Biocenter, Medical University Innsbruck, Innsbruck, Austria*

^3^*Institute of Molecular and Translational Medicine, Faculty of Medicine and Dentistry, Palacky University, Olomouc, Czech Republic*

*^4^Institute of Pharmacy, Pharmaceutical Chemistry, University of Innsbruck, Innsbruck, Austria*

^5^*Research and Development Division, Thailand Institute of Nuclear Technology, Nakhonnayok, Thailand*

*Corressponding author:*

Clemens Decristoforo, *e-mail:* [clemens.decristoforo@i-med.ac.at](mailto:clemens.decristoforo@i-med.ac.at)

Department of Nuclear Medicine, Medical University Innsbruck, Anichstrasse 35, 6020, Innsbruck, Austria

***General***

All commercially available chemicals were obtained as analytical grade and used without further purification. Coupling reagents 1-hydroxy-7-azabenzotriazole (HOAt) and *O*-(7-azabenzotriazol-1-yl)-*N,N,N´,N´*-tetramethyl uroniumhexafluorophosphate (HATU) were purchased from GenScript Corporation (Piscataway, NJ, USA). 9-Fluorenylmethoxycarbonyl (Fmoc) protected glycine (Fmoc-gly-OH) was obtained from Novabiochem (La Jolla, CA, USA). All other chemicals were purchased from Sigma-Aldrich Handels GmbH (Vienna, Austria) or VWR International GmbH (Vienna, Austria).

Analytical reversed-phase high performance liquid chromatography (RP-HPLC) was performed with an UltiMate 3000 UHPLC pump, an UltiMate 3000 autosampler, an UltiMate 3000 column compartment, an UltiMate 3000 variable wavelength detector (Thermo Fisher Scientific, Vienna, Austria) and a GabiStar radiometric detector (Raytest GmbH, Straubenhardt, Germany). An ACE 3 C18, 3 µm 100 Å, 150 x 3.0 mm column (ACE, Aberdeen, UK) with a flow rate of 0.6 ml/min and UV detection at 220 nm or 450 nm were employed. Acetonitrile (ACN)/H_2_O/0.1% trifluoroacetic acid (TFA) was used as mobile phase with the following multistep gradients: 0–1.0 min 10% ACN, 1.0–10.0 min 10–30% ACN, 10.0–11.0 min 30–60% ACN, 11.0–13.0 min 60% ACN (gradient A); 0–1.0 min 10% ACN, 1.0–12.0 min 10–60% ACN, 12.0–14.0 min 60% ACN (gradient B); and 0–1.0 min 10% ACN, 1.0–9.0 min 10–60% ACN, 9.0–10.0 min 60% ACN (gradient C).

Preparative RP-HPLC was carried out on a Gilson 322 HPLC pump with a Gilson UV/VIS-155 detector and a PrepFC automatic collector (Gilson International B.V., Limburg, Germany). A Eurosil Bioselect Vertex Plus, C_18A_ 5*μ*m 300 Å, 30 × 8 mm precolumn and a Eurosil Bioselect Vertex Plus, C_18A_ 5*μ*m 300 Å, 300 × 8 mm column (Knauer, Berlin, Germany) were employed with a flow rate of 2 ml/min and UV detection at 220 nm. The mobile phase ACN/H_2_O/0.1% TFA was used with several multistep gradients.

Matrix-assisted laser desorption/ionization-time of flight mass spectrometry (MALDI-TOF MS) was carried out on a Bruker microflex MALDI-TOF mass spectrometer (Bruker Daltonik, Bremen, Germany) using *α*-cyano-4-hydroxy-cinnamic acid as matrix. Samples were applied on a microScout target (MSP96, Bruker Daltonik) by dried droplet method. All spectra were recorded by summarizing 800 laser shots from reflection acquisition mode with positive ion source. Data processing was performed by Flex Analysis 3.0 software.

***Synthesis***

***Acetylation of [Fe]FSC***

Amino group(s) of [Fe]FSC was acetylated to give acetyl[Fe]FSC ([Fe]MAFC), diacetyl[Fe]FSC ([Fe]DAFC) and triacetyl[Fe]FSC ([Fe]TAFC) which were then used as the starting material of further syntheses. Briefly, to [Fe]FSC (22.1 mg, 28.2 µmol) dissolved in 1 ml MeOH, acetic anhydride (Ac_2_O) (115.8 mg, 1.1 mmol) was added and the resulting mixture was stirred at RT for 5 min. Hereafter acetylation products were isolated by preparative RP-HPLC and dried by lyophilization.

***Tripropanoyl[Fe]FSC*  *([Fe]TPFC)***

Propanoyl chloride (30.6 mg, 330.1 µmol) diluted in 100 µl dimethylformamide (DMF) was added to [Fe]FSC (8.58 mg, 11.0 µmol) dissolved in 0.5 ml DMF. The reaction was carried out at RT under basic condition, pH 10-11, using *N,N*-diisopropylethylamine (DIPEA) as base. After stirring for 10 min, DMF was removed under vacuum and crude product was purified via preparative RP-HPLC.

***Tributanoyl[Fe]FSC* *([Fe] TBuFC)***

To 0.5 ml DMF solution of [Fe]FSC (4.3 mg, 5.5 µmol), butanoyl chloride (10.5 mg, 98.8 µmol) in 100 µl DMF was added. The reaction mixture was alkalized to pH 10-11 using DIPEA and stirred at RT for 10 min. Thereafter, solvent was evaporated and residual was separated by preparative RP-HPLC.

***Acylation of [Fe]DAFC***

The derivatives of [Fe]DAFC with different acyl compounds (propanoyl, butanoyl, benzoyl) were synthesized by acylation at NH_2_ moiety. [Fe]DAFC (3.7 mg, 4.3 µmol) dissolved in 0.5 ml DMF was reacted with propanoyl chloride (6.4 mg, 69.1 µmol), butanoyl chloride (4.1 mg, 38.6 µmol) or benzoyl chloride (5.5 mg, 38.8 µmol). The reaction was carried out at RT under basic condition (pH 10–11). The reaction was complete within 1 h confirmed by RP-HPLC. Thereafter DMF was reduced *in vacuo* and the product was purified via preparative RP-HPLC. Propanoyl[Fe]DAFC ([Fe]DAPFC), butanoyl[Fe]DAFC ([Fe]DABuFC) or benzoyl[Fe]DAFC ([Fe]DABzFC) was collected and finally lyophilized.

***Succinyl Substitution***

To introduce negative charge(s) into [Fe]DAFC, [Fe]MAFC and [Fe]FSC via amino group(s) of FSC, succinic anhydride was used as the reactant. [Fe]DAFC (3.7 mg, 4.3 µmol), [Fe]MAFC (3.9 mg, 4.7 µmol) or [Fe]FSC (4.0 mg, 5.1 µmol) was dissolved in DMF and reacted with an excess of 2, 4 or 6 equiv of succinic anhydride, respectively. DIPEA was added to adjust pH to 10–11 and the reaction mixture was then stirred at RT for 5 min. The solution was concentrated under vacuum and the purification was performed on preparative RP-HPLC. The succinyl[Fe]DAFC ([Fe]DAFC(suc)), disuccinyl[Fe]MAFC ([Fe]MAFC(suc)_2_) or trisuccinyl[Fe]FSC ([Fe]FSC(suc)_3_) was obtained and dried by lyophilization.

***Glycyl Substitution***

Addition of positive charge(s) to [Fe]DAFC, [Fe]MAFC and [Fe]FSC was accomplished by the reaction of corresponding siderophore with glycine amino acid via *in situ* activation of HOAt/HATU. Firstly, the coupling reagents HOAt and HATU were mixed with Fmoc-gly-OH in DMF in the mole ratio of 4:4:3. Then the mixture with an excess of 1.5, 3, or 4.5 equiv of Fmoc-gly-OH was transferred to 1 equiv of [Fe]DAFC (4.2 mg, 4.8 µmol), [Fe]MAFC (5.02 mg, 6.1 µmol) or [Fe]FSC (4.1 mg, 5.2 µmol), respectively. DIPEA was added to adjust pH to 10-11 and the reaction mixture was stirred at RT for 10 min. After solvent removal, subsequent deprotection of Fmoc group was carried out by reacting with 10% piperidine/DMF (v/v) at RT for 2 h. Hereon solvent was reduced, and deprotected product was purified by preparative RP-HPLC. Finally glycyl[Fe]DAFC ([Fe]DAFC(gly)), diglycyl[Fe]MAFC ([Fe]MAFC(gly)_2_) or triglycyl[Fe]FSC ([Fe]FSC(gly)_3_) was received in solid form by lyophilization.

***Demetalation***

Iron-free siderophores were obtained by reacting [Fe]siderophore with 100-fold molar excess of disodium ethylenediaminetetraacetic acid (Na_2_EDTA) solution (50 mM, pH 4) at RT for 2 h. Hereafter, the solution was diluted with H_2_O and then injected directly to preparative RP-HPLC for purification. After solvent removal and lyophilization, siderophore was obtained as white solid: FSC; MAFC; DAFC; TAFC; TPFC; TBuFC; DAPFC; DABuFC; DABzFC; DAFC(suc); MAFC(suc)_2_; FSC(suc)_3_; DAFC(gly); MAFC(gly)_2_; and FSC(gly)_3_.

**Supplemental Table 1.** Found masses, chemical yields and retention time synthesized of siderophores and [^68^Ga]siderophores

| Siderophore | exact mass (calcd.) | | [M+Na]^+^/*[M+H]^+^* | | % yield | | *t_R_* (min) | | [^68^Ga]siderophore |
| --- | --- | --- | --- | --- | --- | --- | --- | --- | --- |
|  | +[Fe] | -[Fe] | +[Fe] | -[Fe] | +[Fe] | -[Fe] | +[Fe] | -[Fe] | *t_R_* (min) |
| FSC | 779.63 | 726.81 | *780.55* | 750.22 | *NA* | 55 | 6.32*^a^* | 8.05*^a^* | 4.77*^c^* |
| MAFC | 821.67 | 768.85 | 844.65 | 792.21 | 8 | 58 | 7.80*^a^* | 9.43*^a^* | 5.66*^c^* |
| DAFC | 863.71 | 810.89 | 886.68 | 834.17 | 32 | 57 | 9.36*^a^* | 10.55*^a^* | 6.37*^c^* |
| TAFC | 905.74 | 852.92 | 928.67 | 876.09 | 25 | 67 | 10.73*^a^* | 11.64*^a^* | 6.98*^c^* |
| TPFC | 947.82 | 895.00 | *948.73* | 918.09 | 52 | 63 | 9.28*^b^* | 9.67*^b^* | 8.01*^c^* |
| TBuFC | 989.90 | 937.08 | *990.90* | 960.29 | 53 | 54 | 10.89*^b^* | 11.29*^b^* | 9.21*^c^* |
| DAPFC | 919.77 | 866.95 | *920.33* | 889.98 | 65 | 61 | 8.36*^b^* | 8.85*^b^* | 7.33*^c^* |
| DABuFC | 933.80 | 880.98 | 956.39 | 904.00 | 53 | 50 | 8.96*^b^* | 9.38*^b^* | 7.74*^c^* |
| DABzFC | 967.81 | 914.99 | *968.32* | 938.01 | 57 | 53 | 10.03*^b^* | 10.27*^b^* | 8.58*^c^* |
| DAFC(suc) | 963.78 | 910.96 | *964.96* | 934.40 | 55 | 59 | 7.90*^b^* | 8.33*^b^* | 6.93*^c^* |
| DAFC(gly) | 920.76 | 867.94 | *921.49* | *869.05* | 51 | 61 | 7.06*^b^* | 7.53*^b^* | 6.46*^c^* |
| MAFC(suc)_2_ | 1021.82 | 968.99 | *1022.311* | 992.44 | 58 | 60 | 7.87*^b^* | 8.25*^b^* | 6.89*^c^* |
| MAFC(gly)_2_ | 935.77 | 882.95 | *937.08* | *883.56* | 44 | 56 | 6.33*^b^* | 7.19*^b^* | 5.86*^c^* |
| FSC(suc)_3_ | 1079.85 | 1027.03 | *1081.05* | 1050.43 | 64 | 58 | 7.85*^b^* | 8.16*^b^* | 6.86*^c^* |
| FSC(gly)_3_ | 950.79 | 897.97 | *951.47* | *898.47* | 38 | 43 | 5.99*^b^* | 6.61*^b^* | 5.35*^c^* |

*^a^* = gradient A, *^b^* = gradient B, *^c^* = gradient C

**Supp. Fig. 1** *In vitro* uptake of [^68^Ga]siderophores in *A. terreus* *wildtype* strain, which lacks the MirB transporter, under iron-starvation condition at 37 °C for 45 min. The uptakes are displayed as % uptake of total activity added (*n* = 3). Uptake under iron-starvation condition with TAFC blocking and in iron-replete condition were < 0.5 % of total activity added except for [^68^Ga]FSC (28 % and 2 %, respectively) (data not shown).
